# Supplementary material for: Key Factors Affecting the Flesh Flavor Quality and the Nutritional Value of Grass Carp in Four Culture Modes
Source: Foods. 2021 Sep 2;10(9):2075. doi: 10.3390/foods10092075 (PMC8471861; doi:10.3390/foods10092075)
Supplement: Supplementary file 1 [file foods-10-02075-s001.zip › foods-1347913-supplementary.pdf]

Table S1. The concentration of amino acids

Amino acids	Control (g/100g)	FBT (g/100g)	GPT (g/100g)	WWT (g/100g)
Aspartate	0.00045	0.00037	0.00056	0.00059
Threonine	0.017	0.010	0.011	0.019
Serine	0.0043	0.0042	0.0047	0.0056
Glutamate	0.0053	0.0036	0.0066	0.0046
Glycine	0.033	0.020	0.026	0.033
Alanine	0.023	0.013	0.016	0.020
Valine	0.0036	0. 0028	0.0047	0.0044
Methionine	-	-	0.0023	-
Isoleucine	0.0015	0.0017	0.0031	0. 0022
Leucine	0.0029	0.0032	0.0050	0.0038
Tyrosine	0.0033	0.0030	0.0036	0.0040
Phenylalanine	-	0.0027	0.0032	0.0032
Lysine	0.026	0.029	0.018	0.033
Histidine	0.081	0.023	0.019	0.046
Arginine	0.013	0.011	0.0064	0.016
Proline	0.025	0.0047	0.0055	0.021

Table S2. The volatile compounds of the control

Muscle sample			Skin sample		
Compounds	Relative content (%)	Retention time (min)	Compounds	Relative content (%)	Retention time (min)
2-Amino-5-methylbenzoic acid	1.32	5.162	Pentanal	0.84	1.701
Benzaldehyde	5.36	6.714	Hexanal	1.56	2.846
1-Octen-3-ol	0.99	7.086	1-Butanamine, N-butyl-	2.39	6.686
4-Acetamidobutyric acid	1.20	7.217	1-Octen-3-ol	21.11	7.231
7H-Dibenzo[b,g]carbazole, 7-methyl-	2.53	7.707	trans-4-Dimethylamino-4'-methoxychalcone	4.09	7.700
Eucalyptol	1.84	8.431	3,6-Bis(N-dimethylamino)-9-ethylcarbazole	4.12	7.817
1-Octanol	3.43	9.513	1,3-Hexadiene, 3-ethyl-2-methyl-	0.90	8.424
Nonanal	23.67	10.410	1-Octanol	9.81	9.500
1-Decene	1.59	12.078	5-Nonanone, 2,8-dimethyl-	1.37	10.134
Decanal	2.68	12.920	Nonanal	8.12	10.417
Nonanoic acid	2.76	14.457	5H-Naphtho[2,3-c]carbazole	0.79	10.782
Formamide, N,N-dibutyl-	0.85	15.209	Formamide, N,N-dibutyl-	9.17	15.188
Carbamodithioic acid, diethyl-, methyl ester	1.21	16.898	Corynan-17-ol, 10-methoxy-	0.81	15.554
Dimethyl phthalate	0.92	18.498	Carbamodithioic acid, diethyl-, methyl ester	6.93	16.898
Butylated Hydroxytoluene	1.06	18.850	2,5-Cyclohexadiene-1,4-dione, 2,6-bis(1,1-dimethylethyl)-	0.73	18.753
Phenol, 2,4-bis(1,1-dimethylethyl)-	3.52	19.670	Phenol, 3-(1,1-dimethylethyl)-4-methoxy-	1.92	18.850
Heptadecane	1.84	23.249	Dihydrocoumarin, 4,4,5,7,8-pentamethyl	15.16	19.015
Tetradecanoic acid	0.82	24.331	Phenol, 2,6-bis(1,1-dimethylethyl)-4-(1-methylpropyl)-	0.90	21.629
Benzene, 1,1'-[1,2-ethanediylbis(oxy)]bis-	0.84	25.131	Benzenamine, N-[4-(1-methylethyl)benzylidene]-4-(1-pyrrolidylsulfonyl)-	4.37	26.669
Hexadecanal	2.90	25.324	1-Hexadecanamine, N,N-dimethyl-	2.53	26.786
2,4,6-Trimethylmandelic acid	21.12	26.497	1,2-Benzenediol, 3,5-bis(1,1-dimethylethyl)-	1.38	33.751
Benzenamine, N-[4-(1-methylethyl)benzylidene]-4-(1-pyrrolidylsulfonyl)-	2.82	26.683	Benzene, 1,2,3,5-tetramethyl-4,6-dinitro-	0.98	35.750
n-Hexadecanoic acid	7.10	27.731			
9-Octadecenoic acid, (E)-	0.86	30.454			
2-Propen-1-one, 1-(4-aminophenyl)-3-phenyl-	1.58	31.599			
Acetamide, 2-[4-(1-oxo-3-phenyl-2-propenyl)phenyloxy]-	0.88	33.750			
Eicosane	1.26	35.516			
Octacosane	0.84	38.281			

Table S3. The volatile compounds of FBT mode

Muscle sample			Skin sample		
Compounds	Relative content (%)	Retention time (min)	Compounds	Relative content (%)	Retention time (min)
Hexanal	16.77	2.915	sec-Butylamine	0.52	1.046
1-Butanol	2.40	4.321	Hexanal	1.52	2.839
Benzene, 1-isocyanato-3-methyl-	1.39	5.107	Heptanal	1.36	5.066
Benzaldehyde	4.59	6.748	2-Heptenal, (E)-	0.93	6.452
1-Octen-3-ol	5.58	7.079	1-Octen-3-ol	5.18	7.080
2,5-Octanedione	5.66	7.224	Furan, 2-pentyl-	2.34	7.383
2-Octenal, (E)-	2.48	9.210	Octanal	4.22	7.693
Cycloheptane	6.27	9.534	1,3-Hexadiene, 3-ethyl-2-methyl-	0.98	8.410
1-Methyl-3-phenylindole	2.90	9.893	3,5-Octadien-2-ol	2.13	8.741
Nonanal	17.05	10.403	2-Octenal, (E)-	3.39	9.134
7H-Dibenzo(a,g)carbazole	1.39	10.810	2-Octen-1-ol, (E)-	1.14	9.410
2-Dodecene, (Z)-	0.88	12.072	1-Octanol	4.28	9.486
Thiazole, 2-ethyl-4,5-dimethyl-	0.96	14.719	5-Hepten-3-one, 5-ethyl-2-methyl-	3.02	10.100
Cyclopentaneethanol, 2-(hydroxymethyl)-.beta.,3-dimethyl-	1.07	14.968	Nonanal	15.96	10.362
Hexadecanoic acid, (2-pentadecyl-1,3-dioxolan-4-yl)methyl ester	0.81	15.071	2-Nonenal, (E)-	5.96	11.761
Formamide, N,N-dibutyl-	1.86	15.188	4-Ethylcyclohexanol	1.29	12.603
Furan, 2,3-dihydro-4-methyl-	1.01	16.064	Decanal	0.66	12.892
Perhydrophenalene, (3a.alpha., 6a.alpha., 9a.alpha., 9b.beta.)-	0.77	16.236	2,4-Nonadienal, (E,E)-	2.05	13.085
2-Decenal, (E)-	1.86	16.526	Octane, 4-methyl-	0.91	13.851
Carbamodithioic acid, diethyl-, methyl ester	2.38	16.885	2-Decenal, (E)-	5.96	14.223
3-Pyridinecarboxylic acid, 4-hydroxy-	1.38	17.319	1-Dodecen-3-ol	0.79	14.678
Metacetamol	1.15	17.684	2,4-Dodecadienal, (E,E)-	2.51	14.968
2,5-Cyclohexadiene-1,4-dione, 2,6-bis(1,1-dimethylethyl)-	1.11	18.746	Formamide, N,N-dibutyl-	2.20	15.181
3-tert-Butyl-4-hydroxyanisole	1.67	18.836	2,4-Decadienal, (E,E)-	4.74	15.478
Cyclododecanol	1.53	21.615	1-Iodoundecane	0.83	16.264
Heptadecane	1.42	23.242	2-Cyclohexen-1-ol	9.37	16.540
Oxirane, tridecyl-	2.88	23.511	2-Cyclohexen-1-one	1.63	16.864
Hexadecanal	7.63	25.311	2-Cyclopentene-1-carboxylic acid, 1,2-dimethyl-, ethyl ester	2.97	17.319
9,10-Anthracenedione	0.78	27.786	Metacetamol	0.55	17.691
			Cyclohexane, octyl-	0.73	18.767
			3',5'-Dimethoxyacetophenone	0.84	18.836
			Heptadecane	0.97	23.242
			Tetradecanal	3.59	23.511
			Hexadecanal	3.51	25.318
			n-Hexadecanoic acid	0.96	27.724

Table S4. The volatile compounds of GPT mode

Muscle sample			Skin sample		
Compounds	Relative content (%)	Retention time (min)	Compounds	Relative content (%)	Retention time (min)
2-Hexanamine, 4-methyl-	2.68	1.046	Pyridine	1.07	2.549
Pyridine	1.43	2.487	Hexanal	2.92	2.818
Hexanal	7.79	2.915	Benzeneethanamine, N,N-dimethyl-	0.68	4.770
Hexyl chloroformate	2.26	4.314	Heptanal	0.57	5.018
Benzaldehyde	7.72	6.735	Benzaldehyde	0.78	6.735
1-Octen-3-ol	2.63	7.079	1-Octen-3-ol	16.17	7.259
Nonane, 2,2,4,4,6,8,8-heptamethyl-	3.15	7.224	Octanal	2.39	7.673
1-Octanol	5.82	9.548	trans-4-Dimethylamino-4'-methoxychalcone	5.26	7.845
Phenol-d6-	0.83	10.169	1-Hexanol, 2-ethyl-	3.71	8.507
Nonanal	8.67	10.403	Hexane, 1-nitro-	3.28	8.886
4-(4-Chlorophenyl)-2,6-diphenylpyridine	1.03	10.982	2-Cyclohexen-1-ol	0.80	9.210
1-Decene	1.31	12.079	1-Octanol	3.14	9.486
Thebacon	1.09	14.388	3-Octanone, 2-methyl-	0.91	10.141
Formamide, N,N-dibutyl-	5.04	15.181	Nonanal	16.70	10.424
2(3H)-Furanone, dihydro-5-propyl-	0.81	16.505	2-Decanone	1.34	12.610
Carbamodithioic acid, diethyl-, methyl ester	6.61	16.885	Decanal	0.60	12.913
p-Isopropoxyaniline	8.07	17.691	Benzothiazole	0.64	13.285
Dimethyl phthalate	1.34	18.484	2-Octen-1-ol, 3,7-dimethyl-	0.62	13.437
Phenol, 3-(1,1-dimethylethyl)-4-methoxy-	3.63	18.843	2,6-Octadien-1-ol, 3,7-dimethyl-, (E)-	1.28	14.058
Oxirane, tridecyl-	1.75	23.511	Formamide, N,N-dibutyl-	10.91	15.175
Hexadecanal	20.01	25.317	3-Piperidinone, 1-ethyl-	0.67	16.057
Cyclohexadecane	2.42	26.379	Carbamodithioic acid, diethyl-, methyl ester	7.80	16.885
Thiocyanic acid carbazol-3,6-diyl ester	2.48	26.676	Tetradecane	0.60	17.312
9,10-Anthracenedione	1.42	27.786	Metacetamol	1.21	17.684
			Dimethyl phthalate	0.68	18.484
			2,5-Cyclohexadiene-1,4-dione, 2,6-bis(1,1-dimethylethyl)-	0.66	18.739
			Dihydrocoumarin, 4,4,5,7,8-pentamethyl	7.98	19.001
			Benzoic acid, 3,4,5-trimethoxy-2-nitro-, methyl ester	0.57	21.677
			2-Methyl-7-phenylindole	0.73	23.801
			Tetradecanal	1.88	25.311
			Anthrone	1.28	27.469
			n-Hexadecanoic acid	0.63	27.690
			9,10-Anthracenedione	0.71	27.772
			dl-2-Ethylhexyl chloroformate	0.84	35.550

Table S5. The volatile compounds of WWT mode

Muscle sample			Skin sample		
Compounds	Relative content (%)	Retention time (min)	Compounds	Relative content (%)	Retention time (min)
Butanoic acid, 3-amino-2-methyl-	2.07	1.060	sec-Butylamine	1.05	1.046
Hexanal	3.14	2.846	Pyridine	1.13	2.218
Hexyl chloroformate	9.27	4.315	1-Hexene	0.72	4.335
2-Amino-5-methylbenzoic acid	1.65	5.114	Heptanal	0.89	5.107
Benzaldehyde	0.71	6.549	1-Butanamine, N-butyl-	3.15	6.700
1-Octen-3-ol	9.28	7.093	Hexane, 3-ethyl-4-methyl-	1.16	6.935
2,3-Octanedione	13.18	7.224	1-Octen-3-ol	24.79	7.217
4-(Anisylideneamino)-cinnamic acid	4.88	7.845	1-Hexanol, 2-ethyl-	1.49	8.410
2-Pentenal, (E)-	1.58	8.493	Benzeneacetaldehyde	0.71	8.810
2-Piperidinone	0.79	9.265	2-Octenal, (E)-	1.75	9.224
1-Octanol	5.91	9.507	1-Octanol	6.21	9.507
Furan, 2,5-dihydro-2,5-dimethoxy-	4.77	10.169	2-Pentene, 4,4-dimethyl-, (E)-	0.74	10.155
Nonanal	8.44	10.438	Nonanal	10.03	10.410
Benzaldehyde, 2,5-bis[(trimethylsilyl)oxy]-	1.39	10.782	7H-Dibenzo(a,g)carbazole	1.33	10.775
Cycloheptane	0.85	12.092	1-Octene, 6-methyl-	0.64	12.092
trans-4-(2-(5-Nitro-2-furyl)vinyl)-2-quinolinamine	1.78	13.616	Tributylamine	1.89	12.589
4-(4-Chlorophenyl)-2,6-diphenylpyridine	1.55	14.389	Decanal	0.58	12.913
Decane, 2,3,4-trimethyl-	0.70	14.733	Formamide, N,N-dibutyl-	11.91	15.181
Formamide, N,N-dibutyl-	2.27	15.202	Furan, 2,3-dihydro-4-methyl-	0.57	16.071
2(3H)-Furanone, dihydro-5-pentyl-	1.30	16.526	Pentanoic acid, 2,2,4-trimethyl-3-hydroxy-, isobutyl ester	0.67	16.271
Carbamodithioic acid, diethyl-, methyl ester	3.83	16.898	Carbamodithioic acid, diethyl-, methyl ester	18.50	16.891
Tetradecane	1.06	17.326	Dodecane	0.73	17.319
Pyridine, 3-butyl-, 1-oxide	0.65	17.705	Metacetamol	0.64	17.691
Benzene, 1-methoxy-4-(methylthio)-	0.70	17.878	3-tert-Butyl-4-hydroxyanisole	2.51	18.843
2,5-Cyclohexadiene-1,4-dione, 2,6-bis(1,1-dimethylethyl)-	1.03	18.753	Benzene, 1-(1,5-dimethyl-4-hexenyl)-4-methyl-	0.64	19.077
9H-Fluorene, 1-methyl-	6.55	18.850	Cyclohexene, 3-(1,5-dimethyl-4-hexenyl)-6-methylene-, [S-(R*,S*)]-	0.71	19.918
Dihydrocoumarin, 4,4,5,7,8-pentamethyl	2.32	19.015	Eicosane	0.63	21.366
1,13-Tetradecadiene	1.04	21.629	Heptadecane	1.13	23.242
Oxirane, tetradecyl-	2.48	23.518	Tetradecanal	2.31	25.317
Tetradecanal	2.61	25.325	1,2-Benzenedicarboxylic acid, butyl 2-methylpropyl ester	0.78	27.765
Heptadecanoic acid	0.64	27.697			
Dibutyl phthalate	0.89	27.772			
Demecolcine	0.66	31.599			
